# In-silico comparison of fungal and bacterial asparaginase enzymes 

**DOI:** 10.22099/mbrc.2024.50123.1981

**Published:** 2024

**Authors:** Negar Tafvizi, Mandana Behbahani, Hassan Mohabatkar

**Affiliations:** Department of Biotechnology, Faculty of Biological Science and Technology, University of Isfahan, Isfahan, Iran

**Keywords:** L-asparaginase, Pseudo amino acid composition, Physicochemical properties

## Abstract

L-asparaginase is a commercial enzyme with a wide variety of applications. Asparaginase is known as an anti-cancer agent that is effective for the treatment of certain lymphomas and leukemias by growth inhibition of human cancer cells. Additionally, asparaginase is used in the food industry in a pretreatment process to decrease the accumulation of carcinogenic acrylamide. In this paper, different aspects of bacterial and fungal asparaginases such as mass, hydrophobicity and hydrophilicity of pseudo amino acid composition (PseAAC), physicochem-ical properties, and structural motifs were studied, and ROC curve statistical analysis was used for the comparison. The results showed that none of the physicochemical properties of fungal and bacterial asparaginase could not be differed, except molecular weight and sequence length. MEME Suite analysis demonstrated that there was a motif that was specific for bacterial asparaginases. However, analysis based on the concept of PseACC indicated a differentiation line between fungal and bacterial asparaginases. In conclusion, although there was not any specific demonstration to separate the bacterial and fungal asparaginases in the case of physicochemical properties, PseAAC analysis can be an appropriate and usable method to differentiate between them.

## INTRODUCTION

L-asparaginase (L-Asparagine amidohydrolase, EC 3.5.1.1) catalyzes the hydrolysis of asparagine amino acid to aspartic acid and ammonia [1]. It can also break down glutamine into ammonia and glutamic acid. Asparaginase has been found in tetrameric, dimeric, hexameric, and monomeric forms when extracted from different sources [2]. In addition, it was identified that there were two isozymes of asparaginase called type 1 and type 2 [3]. However, type 2 has a higher affinity for asparagine than type 1 [4]. Studies have reported the presence of asparaginase in various sources including animals, plants, and microorganisms (bacteria, fungi, algae, and yeasts). In recent years, many papers have focused on this enzyme’s ability because of its biotechnological applications and simplicity of large-scale production [5]. 

The first reason for asparaginas importance is the antineoplastic property that scientists and researchers broadly have observed it strong. Neoplastic cells cannot produce asparagine for their metabolic needs due to the low expression or absence of the asparagine synthetase gene. Therefore, they provide their required asparagine from the surrounding environment. In this regard, asparaginase is an induction chemotherapy standard treatment option against tumor cell growth for acute lymphocytic leukemia (ALL) [2, 5]. The main microbial asparaginase resources for medical approaches are Escherichia sp. and Erwinia sp [1]*. *

L-asparaginase also has a potential role in the food industry as a food processor [6]. Acrylamide is a suspected carcinogen, made in cooked food, and asparaginase is used to reduce acrylamide formation [7]. Asparaginases from diverse sources such as *Aspergillus niger* and* A.* *oryzae* have been assayed in acrylamide reduction in different foods [8].

Regarding asparaginase applications in therapy and biotechnology, there is a problem that should be considered. Prokaryotic resources of asparaginase production can have side effects in long-term therapy including hypersensitivity and immune reactions [9]. To solve this problem, finding new eukaryotic sources of asparaginase production can be a considerable solution.

According to the solution mentioned above, it is important to know which asparaginase source is superior in fungi and bacteria based on physicochemical features. Nowadays, bioinformatics is developed to help researchers with the classification and prediction of various aspects of enzymes to save time and cost. 

Several studies about amino acid sequence and protein structure analysis of enzymes have been reported in the last decade. The amino acid composition of L-asparaginases from fungi, bacteria, and plants was widely studied and compared to differentiate each asparaginase sequence [10-11]. In-silico characterization of fungal asparaginase sequences as well as biochemical characters of the purified enzymes was investigated [12]. Physicochemical properties of endophytic bacterial asparaginase enzymes were characterized by some bioinformatics tools [13]. In the present study, in-silico characterization of fungal asparaginases were compared with bacterial asparaginases for the first time. 

## MATERIALS AND METHODS

**Datasets of L-asparaginase: **Protein sequences of bacterial and fungal asparaginase were selected from NCBI (https://www.ncbi.nlm.nih.gov). 786 and 8342 asparaginase protein sequences from fungi and bacteria, were chosen as our datasets, respectively. To cluster repeated and similar sequences and choose a representative sequence, the CD-HIT program (http://cd-hit.org) and Decrease Redundancy sever from Expasy (https://web.expasy.org) with 95% sequence identity cutoff were employed. The final results included 281 and 229 bacterial and fungal sequences, respectively.

**Physicochemical parameters analysis: **Physicochemical properties of primary protein sequences of fungal and bacterial asparaginases were predicted by ProtParam tool (https://web.expasy.org). ProtParam is available on Expasy website. The properties consisted of sequence length, molecular weight, theoretical PI, aliphatic index, instability index, positively charged, negatively charged, and grand average of hydropathicity (GRAVY).

**Pseudo amino acid compositions (PseACC): **Identifying various features of uncharacterized proteins is one of the most important tasks facing us today in bioinformatics because its obtained information has a significant effect on the improvement of system’s biology and proteomics [14]. That’s why the concept of PseACC was introduced in 2001 [15]. PseACC is based on both amino acid composition and sequence order effect. This method is introduced to extract the features of amino acid composition and physical and chemical characteristics of amino acids [16]. In another word, PseACC is described by a set of 20^+λ^ distinct factors. The 20 part shows the AAC components and the λ part illustrates sequence order correlation [17]. PseACC plays an important role in converting a character sequence of a protein to a numerical sequence. The feature descriptor extraction and model construction were implemented in a machine-learning platform that served as protein sequence analysis and prediction [18]. Different physicochemical parameters like hydrophobicity, hydrophilicity, and side chain mass are the factors to make such a conversion [19]. 

In this study, BioSeq-Analysis 2.0 web server (http://bliulab.net/BioSeq-Analysis2.0/) was utilized to achieve the numerical sequence. Four machine learning algorithms with diverse component modes were analyzed. The first machine learning algorithm was support vector machine; a powerful classification method based on the idea of the generalized linear classifier [20]. OET-KNN algorithm was the second one, worked based on the Dempster-Shafer theory. Each neighbor in a pattern was classified as evidence supporting certain hypotheses [21]. Another machine was Random Forest which represented randomly generated trees, predicted different classes as a training dataset, and then the class with a greater predicted number among the trees was implemented as the test data [22]. The last platform was Covariance Discriminant machine-learning. This analysis was the formulation of classifying rules based on multiple training datasets which are classified by those determined rules [23]. The results of the assessment were evaluated via five parameters: ACC (Overall accuracy), MCC (Mathews Correlation Coefficient), AUC (Area under the curve), Sn (Sensitivity) and Sp (Specificity). ACC, MCC, Sn and Sp were calculated referring to Eqs (1-4) [17].

1. Acc = (TP+TN)/(TP+TN+FP+FN)
2. MCC = ((TP*TN) – (FP*FN))/√((TP+FP) (TP+FN) (TN+FP) (TN+FN))
3. Sn = TP/(TP+FN)
4. Sp = TN/(TN+FP)

TP, TN, FP and FN are, respectively short form of True Positive, True Negative, False Positive and False Negative. 

**Secondary structure analysis: **It is proved that structural information provides insight into protein function [24]. In this regard, we can employ automatic database search methods to design drugs and understand more details of protein-protein interaction networks [25]. For predicting the secondary structure of the protein, the Garnier-Osguthorpe-Robson IV (GOR IV) online tool (https://npsa-prabi.ibcp.fr/) was used [26] ,and Alpha helix, extended strand, and random coiled were predicted.

**Motif discovery: **The conserved motifs in bacterial and fungal asparaginase were predicted by the MEME Suite web server (http://meme.nbcr.net). Analysis of motif sequences represents features like DNA binding sites and protein interaction domains [27]. For predicting domains and the family relations of characterized motifs, InterPro (https://www.ebi.ac.uk) was employed [28]. Here, conserved motifs in both bacterial and fungal asparaginases were compared.

**Statistical analysis: **Statistical tests were introduced to compare two or more different diagnostic systems [29]. To compare amino acid composition and secondary structure physicochemical properties of asparaginases between bacteria and fungi sequences, Receiver Operator Characteristic (ROC) curve analysis (http://melolab.org/star/roc_analysis.php) was used. Accuracy (ACC) is the parameter that describes a binary classification. The acceptable classification performance is described when ACC is more than 80%. Bio-seq Analysis 2.0 server also uses the ROC analysis system for comparison. 

## RESULTS

To evaluate the results of ProtParam server, ROC curve was used (Table 1). Two parameters, sequence length and molecular weight, showed the differences between bacterial and fungal enzymes receiving more than 80% accuracy. However, other physiochemical properties did not show significant differences. In addition, the frequency of all 20 amino acids in bacterial and fungal asparaginases was determined (Table 2). ROC curve indicated that bacterial asparaginases could be distinguishable from fungal asparaginases in aspect of amino acid frequency such as glutamic acid, glycine, cysteine, and serine.

**Table 1:** ROC curve analysis of ProtParam results

**Physico-chemical parameters**	**ACC**
Theoretical PI	0.6503
Instability index	0.6523
**Sequence length**	**0.9198**
**Molecular weight**	**0.8959**
Aliphatic index	0.6405
GRAVY	0.5658

(The acceptable results were shown in bold)

**Table 2:** ROC analysis of amino acids frequency between bacterial and fungal asparaginases

**Amino acids**	**ACC**
Alanine	0.7390
Arginine	0.7217
Asparagine	0.5797
Aspartic acid	0.6027
**Cysteine**	**0.8503**
Glutamine	0.5739
**Glutamic acid**	**0.8292**
**Glycine**	**0.8484**
Histidine	0.6679
Iso leucine	0.7044
leucine	0.7447
Lysine	0.6833
Methionine	0.5854
Phenyl alanine	0.7639
Proline	0.6718
**Serine**	**0.8292**
Threonine	0.6180
Tryptophan	0.4395
Tyrosine	0.6641
Valine	0.6814

(The acceptable results were shown in bold)

For analyzing the results of PseACC, four types of machine learning algorithms while considering three physicochemical parameters (mass, hydrophobicity, hydrophilicity), were used (Table 3). The results showed that bacterial and fungal Asparaginase sequences were significantly different. The highest performance was achieved by OET-KNN machine-algorithm (ACC 99.06%).

The results of secondary structure prediction were evaluated using ROC curve analysis (Table 4). In the case of the secondary structure prediction, the results exhibited that bacterial Asparaginases were not significantly different from fungal asparaginases. ACC values of all three parameters were less than 80%.

**Figure 1:**
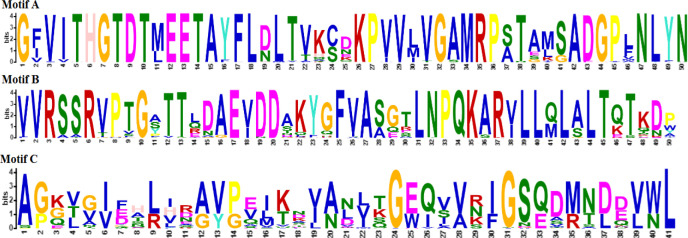
The most probable motifs in bacterial sequences**. Motif A. **50 amino acids; Family type: Asparaginase/glutaminase-like; Domain type: Asparaginase, N-terminal; Homologous Superfamily: Asparaginase/ glutaminase-like, Asparaginase, N-terminal domain superfamily; Active site type: Asparaginase/glutaminase, active site 2. **Motif B. **50 amino acids; Family type: Asparaginase/glutaminase-like; Domain type: Asparaginase, C-terminal; Homologous Super Family: Asparaginase/glutaminase-like, Asparaginase, C-terminal.** Motif C. **41 amino acids, Family type: Asparaginase/glutaminase-like; Homologous Superfamily: Asparaginase, N-terminal domain superfamily.

**Figure 2:**
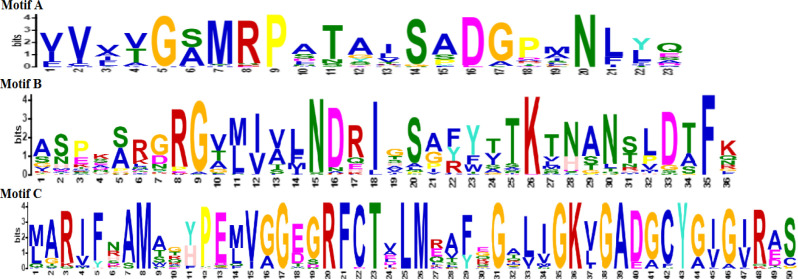
The most probable motifs in fungal sequences**. Motif A. **23 amino acids, Family type: Asparaginase/ glutaminase-like; Domain type: Asparaginase, N-terminal; Homologous superfamily: Asparaginase/glutaminase-like. **Motif B. **36 amino acids, Family type: Asparaginase/glutaminase-like; Domain type: Asparaginase, N-terminal; Homologous superfamily: Asparaginase/glutaminase-like.** Motif C. **50 amino acids, Family type: Asparaginase-II.

MEME suite server highlighted three bacterial (Motif A, B, and C) and three fungal asparaginase motifs (Motif A, B, and C). InterPro server expressed family relations and length of bacterial and fungal asparaginase motifs, which are shown in Figures 1 and 2. On average, fungal motifs were shorter than bacterial ones. Motifs A and B of bacterial asparaginases belonged to the same family type as Motifs A and B of fungal sequences. Motif C of bacterial sequences was a member of asparaginase/glutaminase-like. While fungal Motif C was characterized as a member of the asparaginase II family.

**Table 3:** The results of four different machine-learning algorithm

**Random Forest machine-learning algorithm**	
**ACC**	**0.9891**
**MCC**	0.9777
**AUC**	0.9997±0.0004
**Sn**	0.9858
**Sp**	0.9935
**Support vector machine-learning algorithm**	
**ACC**	**0.9784**
**MCC**	0.9551
**AUC**	0.9957±0.0033
**Sn**	0.9825
**Sp**	0.9724
**Covariance Discriminant machine-learning algorithm**	
**ACC**	**0.9555**
**MCC**	0.9137
**AUC**	0.2904±0.054
**Sn**	0.9235
**Sp**	1.0
**OET-KNN machine-learning algorithm**	
**ACC**	**0.9906**
**MCC**	0.9808
**AUC**	0.9978±0.0028
**Sn**	0.991
**Sp**	0.9901

(The acceptable results were shown in bold)

**Table 4:** ROC analysis of GOR IV server results between bacterial and fungal asparaginase sequences

**parameters**	**ACC**
Alpha helix	0.7894
Extended strand	0.6978
Random coiled	0.6777

Four physicochemical properties (theoretical PI, instability index, aliphatic index, and Gravy) of all three types of enzyme motifs in bacteria and fungi were selected and analyzed by ROC analysis. The results shown in Table 5, indicated that bacterial asparaginase Motif C was different from fungal asparaginase Motif C in the case of aliphatic index, GRAVY, theoretical PI, and instability index. ROC analysis and InterPro results both showed the difference between bacterial and fungal asparaginases in Motif C. Therefore, it was concluded that the results of ROC analysis were correlated with InterPro results. 

**Table 5:** ROC analysis of physicochemical properties of motifs A, B and C

**Physico-chemical properties**	**Motif A (ACC)**	**Motif B (ACC)**	**Motif C (ACC)**
Aliphatic index	0.5135	0.6850	**0.8421**
Instability index	0.3321	03223	**0.8421**
Theoretical PI	0.6679	0.6286	**0.8483**
GRAVY	0.6750	0.6777	**0.8483**

## Discussion

The purpose of this study was to compare bacterial and fungal asparaginase’s different cases. Bacterial asparaginases were compared to fungal asparaginases by computational methods for the first time. In this regard, powerful bioinformatics techniques such as physicochemical properties, secondary structure, PseAAC, and motif analysis tools were used. 

Our results exhibited a separation line between bacterial and fungal asparaginase sequences in some physicochemical parameters. The Molecular weight and sequence length of asparaginase between fungi and bacteria showed a critical difference. Since fungi being eukaryotes, it is expected that fungal asparaginase sequences are heavier, and longer than bacterial sequences, and our efforts showed the same outcome. Amino acid composition analysis of bacterial and fungal asparaginases expressed a significant difference in the frequency of a few amino acids such as glutamic acid, glycine, serine, and cysteine. The Aliphatic index is the parameter that demonstrates the volume of aliphatic side chains (alanine, valine, isoleucine, and leucine) that are present in a protein. The aliphatic index of fungal asparaginase motifs was greater than bacterial asparaginase motifs. Because the aliphatic index could be an effective factor for the increase of thermostability of the protein, fungal asparaginases may be more practical than bacterial asparaginases in industrial applications. Regarding the instability of the protein, if the index is smaller than 40, it means that the protein is stable which is the state that the optimal activity could be seen [30]. Our results showed that there was no difference in the case of stability between fungal asparaginases and bacterial asparaginases. GRAVY is considered as hydropathy value of amino acid composition. When this index is negative, the peptide is hydrophobic [31]. In bacteria, the number of hydrophobic asparaginases was more than in hydrophilic ones. But, in fungi, it was inverted. However, ROC analysis received an accuracy of 56.58%. According to the motif’s investigation outputs, the one and only difference between bacterial and fungal sequences was seen in motif C with a low accuracy (84.21%).

Previous studies have proved that Chou’s PseAAC was a useful technique to classify proteins. In 2017, the dissimilarity between Reverse transcriptase enzyme of HIV-1 and HIV-2 was explained using statistical analysis of PseAAC [32]. In one paper, the result of Lipase enzyme classification between two groups of bacteria and fungi presented over 80% accuracy based on the concept of PseAAC [33]. Sudheer Gupta and his colleagues compared the toxicity of peptides and proteins with various prediction models in the case of PseACC [34]. In 2019, Random Forest machine learning was examined as a model for predicting the difference between toxin and non-toxin peptides of animal [35]. In a published paper in 2020, the PseAAC of Alkaline phosphatase in one class and Acid phosphatase in another class was analyzed [36]. Our results confirmed the efficiency of PseACC technique to achieve more than 95% accuracy regarding three physicochemical features (mass, hydrophobicity, hydrophilicity) for all four machine algorithms. While, in other analyzed severs, there was not such accuracy that was revealed from BioSeq-Analysis server. This represents that using Chou’s PseAAC helps us to predict whether a given asparaginase sequence is related to bacteria or fungi. 

These days, our concern is to find asparaginase from eukaryotic sources with lower toxicity, adverse effects, higher activity levels, and large-scale production to replace bacterial sources usage for therapeutic and industrial purposes. Our bioinformatical analysis showed differences in some ways particularly the BioSeq-Analysis outcomes that surprised us in the way of its accurate precision. In the future, our efforts can go in a way that analyzes more aspects of the asparaginase by computational techniques with higher accuracy.

## References

[1] Darvishi F, Faraji N, Shamsi F (2019). Production and structural modeling of a novel asparaginase in Yarrowia lipolytica. Int J Biol Macromol.

[2] Batool T, Makky EA, Jalal M, Yusoff MM (2016). A comprehensive review on L-asparaginase and its applications. Appl Biochem Biotechnol.

[3] Ohnuma T, Bergel F, Bray RC (1967). Enzymes in cancer: asparaginase from chicken liver. Biochem J.

[4] Campell HA, Mashburn LT, Boyse EA, Old LJ (1967). Two L-asparaginases from Escherichia coli B Their separation, purification, and antitumor activity. Biochemistry.

[5] Souza PM, de Freitas MM, Cardoso SL, Pessoa A, Guerra ENS, Magalhaes PO (2017). Optimization and purification of L-asparaginase from fungi: A systematic review. Crit Rev Oncol Hematol.

[6] Dhanam JG, Kannan S (2013). L-asparaginase-Types, perspectives and applications. Advanced BioTech.

[7] Cachumba JJM, Fernandes Antunes FA, Dias Peres GF, Brumano LP, Dos Santos JC, Da Silva SS (2016). Current applications and different approaches for microbial L-asparaginase production. Braz J Microbiol.

[8] Shi R, Liu Y, Mu Q, Jiang Z, Yang S (2017). Biochemical characterization of a novel L-asparaginase from Paenibacillus barengoltzii being suitable for acrylamide reduction in potato chips and mooncakes. Int J Biol Macromol.

[9] Duval M, Suciu S, Ferster A, Rialland X, Nelken B, Lutz P, Benoit Y, Robert A, Manel AM, Vilmer E, Otten J, Philippe N (2002). Comparison of Escherichia coli–asparaginase with Erwinia-asparaginase in the treatment of childhood lymphoid malignancies: results of a randomized European Organisation for Research and Treatment of Cancer-Children's Leukemia Group phase 3 trial. Blood.

[10] Mousavi SE, Mohabatkar H, Behbahani M (2024). Comparative in silico analysis of fungal and bacterial alkaline serine proteases: Insights into structure, function, and evolution. Iran J Sci.

[11] Dwivedi VD, Mishra SK (2014). In silico analysis of L-asparaginase from different source organisms. Interdiscip Sci.

[12] Elsaba YM, Salama WH, Soliman ERS (2022). In silico and biochemical analysis on a newly isolated Trichoderma asperellum l-asparaginase. Biocatal Agricul Biotech.

[13] Zadeh Hosseingholi E, Neghabi N, Molavi G, Gheibi Hayat SM, Shahriarpour H (2020). In silico identification and characterization of antineoplastic asparaginase enzyme from endophytic bacteria. IUBMB Life.

[14] Chou KC, Shen HB (2009). Recent advances in developing web-servers for predicting protein attributes. Nat Sci.

[15] Chou KC (2001). Prediction of protein cellular attributes using pseudo‐amino acid composition. Proteins.

[16] Su W, Qian X, Yang K, Ding H, Huang C, Zhang Z (2023). Recognition of outer membrane proteins using multiple feature fusion. Front Genet.

[17] Samman N, Mohabatkar H, Rabiei P (2023). Using several pseudo amino acid composition types and different machine learning algorithms to classify and predict archaeal phospholipases. Mol Biol Res Commun.

[18] Huang A, Lu F, Liu F (2023). Discrimination of psychrophilic enzymes using machine learning algorithms with amino acid composition descriptor. Front Microbiol.

[19] Shen HB, Chou KC (2008). PseAAC: a flexible web server for generating various kinds of protein pseudo amino acid composition. Anal Biochem.

[20] Manavalan B, Basith S, Shin TH, Wei L, Lee G (2019). mAHTPred: a sequence-based meta-predictor for improving the prediction of anti-hypertensive peptides using effective feature representation. Bioinformatics.

[21] Sen TZ, Jernigan RL, Garnier J, Kloczkowski A (2005). GOR V server for protein secondary structure prediction. Bioinformatics.

[22] Alballa M, Butler G (2020). Integrative approach for detecting membrane proteins. BMC Bioinformatics.

[23] Boulesteix AL, Janitza S, Kruppa J, König IR (2012). Overview of random forest methodology and practical guidance with emphasis on computational biology and bioinformatics. Wiley Interdiscip Rev Data Min Knowl Discov.

[24] Sajana  OK, Sajesh  TA (2023). Robust quadratic discriminant analysis using Sn covariance. Commun Stat-Simul C.

[25] Rost B (2001). Protein secondary structure prediction continues to rise. J Struct Biol.

[26] Garnier J, Gibrat JF, Robson B (1996). GOR method for predicting protein secondary structure from amino acid sequence. Methods Enzymol.

[27] Bailey TL, Boden M, Buske FA, Frith M, Grant CE, Clementi L, Ren J, Li WW, Noble WS (2009). MEME SUITE: tools for motif discovery and searching. Nucleic Acids Res.

[28] Mulder N, Apweiler R (2007). Interpro and interproscan, in Comparative genomics. Springer.

[29] Hajian-Tilaki K (2013). Receiver operating characteristic (ROC) curve analysis for medical diagnostic test evaluation. Caspian J Intern Med.

[30] Ikai A (1980). Thermostability and aliphatic index of globular proteins. J Biochem.

[31] Lebreton A, Moreau V, Lapalud P, Cayzac C, André S, Nguyen C, Schved JF, Lavigne G, Granier C (2011). Discontinuous epitopes on the C2 domain of coagulation Factor VIII mapped by computer‐designed synthetic peptides. Br J Haematol.

[32] Behbahani M, Mohabatkar H, Nosrati M (2017). Discrimination of HIV-1 and HIV-2 reverse transcriptase proteins using Chou’s PseAAC. Iran J Sci Technol, Trans A: Science.

[33] Behbahani M, Mohabatkar H, Nosrati M (2016). Analysis and comparison of lignin peroxidases between fungi and bacteria using three different modes of Chou’s general pseudo amino acid composition. J Theor Biol.

[34] Gupta S, Kapoor P, Chaudhary K, Gautam A, Kumar R (2013). Open Source Drug Discovery Consortium Raghava GPS In silico approach for predicting toxicity of peptides and proteins. PlOS One.

[35] Pan Y, Wang S, Zhang Q, Lu Q, Su D, Zuo Y, Yang L (2019). Analysis and prediction of animal toxins by various Chou's pseudo components and reduced amino acid compositions. J Theor Biol.

[36] Amoozadeh M, Behbahani M, Mohabatkar H, Keyhanfar M (2020). Analysis and comparison of alkaline and acid phosphatases of Gram-negative bacteria by bioinformatic and colorimetric methods. J Biotech.

